# Inherited, congenital and acquired disorders by hemostasis (vascular, platelet & plasmatic phases) with repercussions in the therapeutic oral sphere

**DOI:** 10.4317/medoral.19560

**Published:** 2013-10-13

**Authors:** Juan J. Arrieta-Blanco, Ricardo Oñate-Sánchez, Federico Martínez-López, Daniel Oñate-Cabrerizo, Maria C. Cabrerizo-Merino

**Affiliations:** 1 Medical Doctor. Stomatologist. Associate Chief of Odontostomatological Service of the Jiménez Díaz-Capio Foundation. Madrid. Autonomic University of Madrid; 2 Medical Doctor. Stomatologist. Permanent Professor of University. Docent Unit of Special Patients. University Odontological Clinic. Faculty of Medicine. Murcia; 3 Medical Doctor. Odontologist. USBD Medical Center of Fuente Álamo and Mazarrón. Contributor of Docent Unit of Special Patients. University Odontological Clinic. Faculty of Medicine. Murcia; 4 Doctor. Contributor of Docent Unit of Special Patients. University Odontological Clinic. Faculty of Medicine. Murcia; 5 Medical Doctor. Stomatologist. USBD Medical Center of Ranero. Associate professor of Docent Unit of Special Patients. University Odontological Clinic. Faculty of Medicine. Murcia

## Abstract

The hemostasis alterations, either congenital or hereditary origin, and acquired, are circumstances that hinder oral care to patients who suffer them and also generates in the professional who has to attend, high stress. Bleeding control once established and dental treatment planning, both in the aspect of preparation, as the realization of the odonto-stomatological therapeutic, has suffered updates that do need to remember certain aspects of the care of these patients. But we must not forget that the hematologist or internist who controls the patient’s medical condition, is a cornerstone for the planning and implementation of treatment plans. We must also remember that, in certain circumstances, treatment should be performed in a hospital setting. In this review, we aim to provide the odonto-stomatologist guidance on how to address the problem and provide simple and updated guidelines to apply in the treatment of these people.

** Key words:**Hemostasis disorder, oral care protocols, haemorrhagic and thrombotic disorders, haemophilia, von willebrand disease, desmopressin, purple, thrombocytopenia, thrombocytopathies.

## Introduction

Hemostasis means the whole body’s physiological processes whose ultimate goal is to prevent blood loss when altering the integrity of the vascular system structures. When this delicate balance is disturbed, can appear both clinical bleeding (haemorrhagic diathesis) and hypercoagulable (thromboembolic syndromes).

Much of the odontostomalogical activity may result oral bleeding without danger to the patient, but sometimes this represents a serious risk when the ability to control bleeding is diminished by alteration in some phase of hemostasis, either congenitally or acquired. These patients may have bleeding gums, characterized by being more persistent than more intense, so the volume of blood loss could be significant. This fact is important because mild or minimal trauma, such as those ones that may happen eating or brushing your teeth, may be sufficient to cause gingival bleeding in these patients (1). It is therefore essential that the stomatologist properly recognize and identify patients at risk of bleeding during dental treatment to prevent or decide what measures to take for bleeding.

In the hemostasis process are different stages and phases, which involved different cell lines and different proteins (soluble in idle status) of blood. The final result is the formation of a red/fibrin mesh (insoluble protein in the blood) inside it encompassed blood cells (platelets, erythrocytes) are found. This grid/mesh acts as a barrier and prevents the loss of blood vessel injury by until the vascular tree is repaired. Before vascular injury in hemostasis, will produce two successive stages, with primary and secondary hemostasis three phases: a) vascular phase b) platelet phase c) plasma phase with plasma proteins involved in coagulation and clot removal later by fibrinolysis.

## I Revision

I) Primary Hemostasis

It’s the primary hemostatic plug formation. Depends on the vascular integrity (endothelium and subendothelium), and platelet function (quantitative and qualitative). During this stage two mechanisms are involved: one vessel and another platelet.

A) Vascular spasm.: This vasoconstrictor response serves two purposes: it reduces blood loss, thanks to the closure of the injured vessel, and starts the second phase, facilitating platelet adhesion, by a change in the electric charge and exposure of the collagen fibers in the injured vascular wall (2), aided by a number of substances and structures that exist in the vascular endothelium (PGI2, ADP-asa, thrombomodulin, tissue Activators Plasminogen and von Willebrand factor, fibronectin, collagen fibers and proteoglycans, etc).

B) Platelet Activation. Platelets are cell fragments, without nucleic acids inside, of the megakaryocytes (3). Inside are two types of granules: a) α granules, round and ovoid. Containing hydrolytic enzymes, fibrinogen, platelet factor 4, clotting factors, trombostenina and other compounds b) dense granules containing serotonin, ADP, ATP, calcium, potassium, thromboxane A2 and substances involved in hemostasis. Platelet membrane is formed by a phospholipid-protein trilaminar membrane, whose inner part filaments communicate with the surface. On the surface of the membrane, appear many glycoproteins that are critical for platelet adhesion and aggregation.

In the platelet plug formation are two stages: Firstly apposition and platelet adhesion and secondly platelet aggregation and secretion (4-6).

II) Secondary Hemostasis

It’s called plasma phase, covering the phenomena of coagulation and fibrinolysis. Recently, it has been proposed a new model in clotting, which describes three phases (initiation phase, amplification phase and propagation phase). In this new model are provided novel concepts as “The Tisular complex factor-F VII” that participates in the activation of factor IX, what means that the intrinsic and extrinsic ways are linked almost from the beginning of the process and also, the full process is not made continuously but is done in three consecutive phases, actively participating in the last two, platelets and thrombin (7). Also of great importance is the recognition of the involvement of the cellular elements (typically not included in this phase), in which membranes and cellular structures numerous enzymatic processes and activation factors are produced equally. They intervene secreting substances and activating factors and their presence is crucial for the formation of complexes of factors with catalytic / accelerator ability of the biochemical phenomenas that occur during the processes of coagulation activation. Last but not least, we must remember that in the plasma phase of hemostasis there are also included anticoagulation systems, which the body uses to maintain the vascular system without narrowing or blockages, which is mediated by protein S, protein C and thrombomodulin at the injury site.

III) Diagnostic tests for the evaluation of the hemostasis.

- Platelet count: The normal levels are between 150,000 and 400,000 cell / mm3

- Morphology and platelet size control.

- Bleeding time: Ivy’s test measures the time in minutes and is usually less than 9.

- Platelet aggregation (8,9) is made by an aggregometer, that allows us to assess the state of platelet function .

- Prothrombin time (PT): Gives information about factors II, V, VII, IX and X and it is between 11-14 s.

- The international normalized ratio (INR): It’s a standardized method and is calculated by dividing the patient’s prothrombin time by the normal or control prothrombin time, and all that, elevated to the ISI value (International Sensitivity Index). Their normal values are between 0.8 -1.2.

- Activated partial thromboplastin time (APTT): Measures the functionality of the intrinsic and common pathway of the coagulation cascade. Normal values vary from 25 to 40 s.

- Thrombin Time (TT): Time that plasma takes to coagulate by adding thrombin. Useful in qualitative and quantitative disorders of fibrinogen, presence of inhibitors of fibrinogen-fibrin conversion and polymerization inhibitors increase. Their normal values are 10 to 15 s.

- Quantification of coagulation factors and activity levels: On one hand, measures the quantity present and secondly the rate of activity of such factors.

## II Hemostasis Patology

1) Primary Hemostasis Alterations: We are going to briefly describe then, the changes in the blood vessels (angiopathy) and platelets.

A) Changes in blood vessels: Vascular disorders are a heterogeneous group of diseases or conditions that are characterized by easy breakage, with consequent bleeding of small vessels (arterioles and capillaries) (10), Vascular purples enrolled usually minor bleeding in the skin, and in them, the coagulation tests and platelet count are usually normal. Vascular diathesis are classified by hereditary / congenital disorders and acquired as shown in  Table 1, being bolded those one that have more interest for the dentist. In processes with altered subendothelial and perivascular tissue either congenitally or not, subcutaneous hemorrhages occur with minimal trauma, and wound healing will be hindered.

**Table 1:**
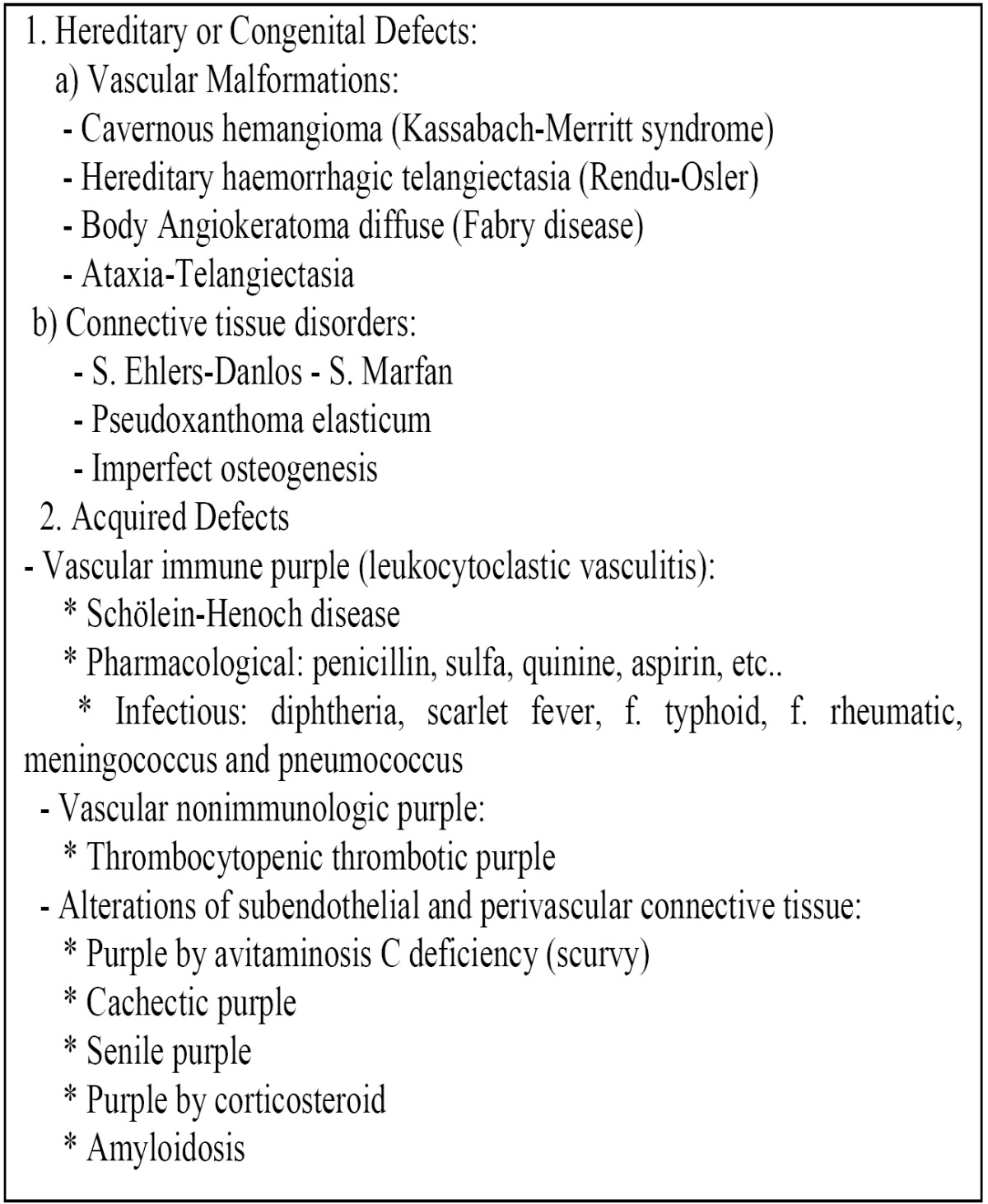
Clinical entities that can provoke alterations in vascular primary hemostasis.

B) Platelet Disorders: inherited platelet disorders are a cause of haemorrhagic syndromes, although rare, ranging from minimal to severe bleeding (11). The clinic is similar in all platelet disorders, studying with a purpuric syndrome of cutaneous-mucosal multiple and spontaneous bleeding. We can distinguish two different types of alterations.

1) Thrombocytopenias: It’s when platelets are below 150.000/mm3. The chances of bleeding when faced a trauma increase when the numbers are between 50.000 and 100.000 / mm3 and if the figure is less than 20.000 / mm3, there’s risk of spontaneous bleeding.  Table 2 describes the various possibilities differentiating between changes in production, increased destruction, alterations in the distribution and idiopathic or hereditary illnesses, also called hereditary thrombocytopenia (thrombocytopenia relatives). In them, there is not a sufficient number of platelets to ensure hemostasis (12). They usually have mild or moderate thrombocytopenia with bleeding little history consistent with platelet counts. Some family thrombocytopenia may also affect platelet morphology and / or function. Of all the above, the most interesting for dentists are marked in bold in  Table 2.

**Table 2:**
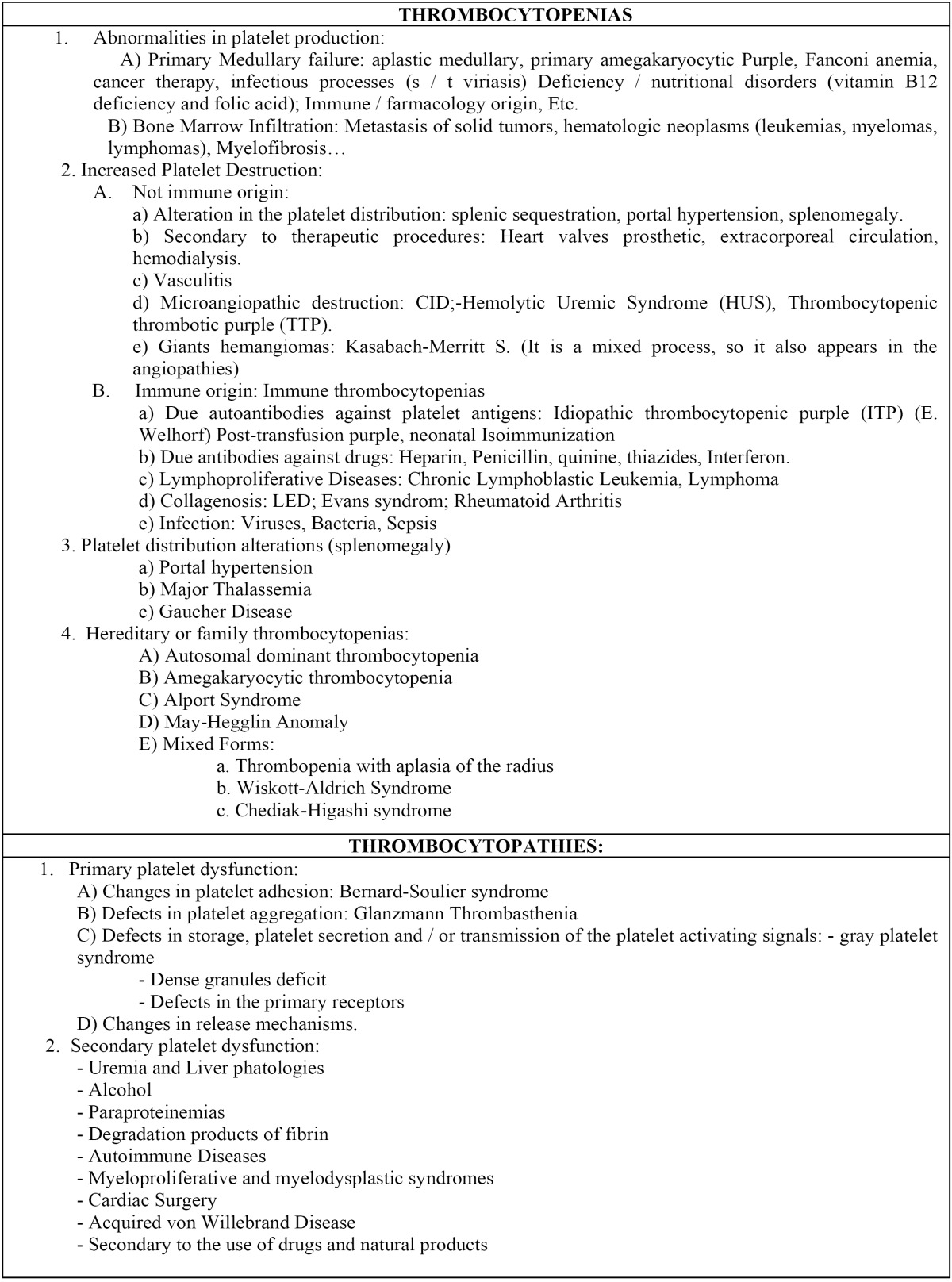
Clinical entities that can result alterations in platelet primary hemostasis.

2) Thrombocytopathies: Functional disorders of platelets (also shown in  Table 2) are generally classified into primary or hereditary disorders and secondary or acquired. Primary abnormalities of platelet function are rare. By contrast, the acquired disorders are very common and are associated with common diseases in clinical practice and treatments administered to patients. Some authors (11), think that primary alterations may be due to defects and / or malfunctions in: the platelet surface glycoproteins, intraplatelets granules; cytoskeletal structural proteins of thrombocytes, catalytic / host / activating activity of the processes of plasmatic phase in the hemostasis or in the transmission systems of messages / signals from the surface to the platelet cytoplasm. Those clinical entities that have more interest for the dentist are marked in bold in  Table 2 too.

2) Alterations in the plasmatic phase (coagulopathies) Plasmatic alterations that cause alterations in the secondary hemostasis process are generically called coagulopathies. Clinically are manifested with severe bruising, but it may also be manifested with bleed hemorrhages in the cutaneous-mucosal level. Alterations may appear both coagulation and fibrinolysis, and according to their origin; they are typically classified as congenital / hereditary and acquired ( Table 3).

**Table 3:**
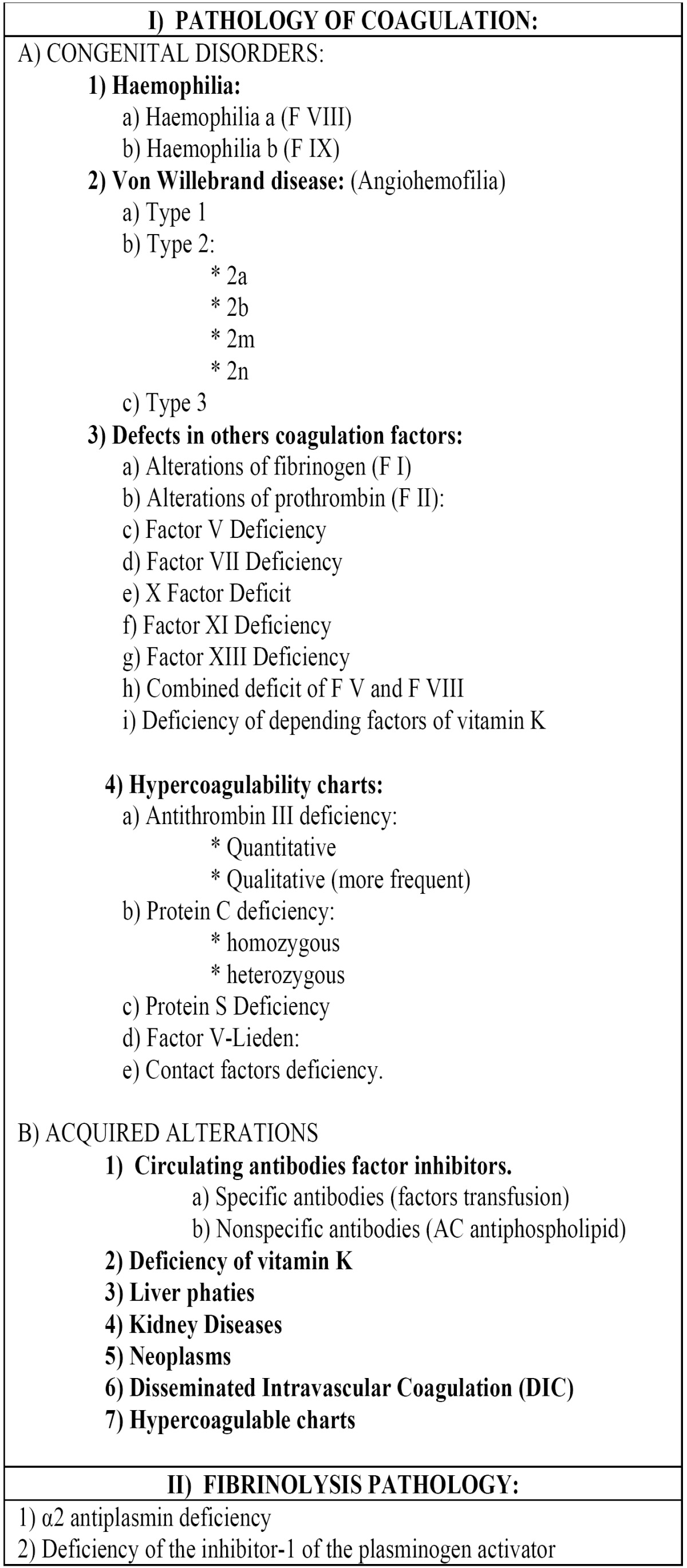
Clinical Entities that may lead to alterations in the plasma phase.

A) Pathology of Bleeding:

1) Congenital disorders: are perhaps the best-known works at the level of the general population and are among others:

a) Haemophilia: There are two different clinical entities. One is haemophilia A, which lacks the factor VIII and constitutes 80-85% of cases. It linked recessive inheritance to chromosome X, although up to 30% of cases are spontaneous mutations. Depending on the degree of activity of the factor is classified (1) into: mild (6-30% factor activity), moderate (1-5% activity), and severe (<1% activity, ie <1 U / dl) with spontaneous bleeding. The normal rates of the factor range between 50 and 100 IU per deciliter (1). The other is haemophilia B, which lacks the F IX and constitutes 16% of cases. Its transmission is sex-linked recessive. Clinically indistinguishable from form A and its severity is also marked by the degree of activity.

b) Von willebrand disease: Also called Angiohemofilia and is due to congenital deficiency VWF variable that can be quantitative or qualitative. It is the most common inherited coagulopathy (13,14), affecting between 1-4% of the world population. The factor is a multimeric and multifunctional molecule that facilitates platelet adhesion after the loss of integrity of the endothelium of the vessels, while the phenomena involved in platelet aggregation and accompanies the factor VIII, transporting it through the bloodstream and protecting of plasma proteases (13). Diagnosis requires the presence of a history of bleeding, reduction in the number or functional impairment of von Willebrand factor and demonstration of its inheritance pattern. It’s divided into three main types (1) shown in  Table 3. VWF deficiency seems an impaired in the primary hemostasis and haemophilia A (especially the kind 3). The number of platelets is normal. Determination of bleeding time has little value in mild cases and is poorly reproducible. APTT may be normal or altered in types 1, 2a and 2b, whereas 2n and 3 are greatly altered. Definitive diagnosis gives VWF quantification by immunological methods. Appears muco-cutaneous bleeding and sometimes, delayed bleeding (in days) after extractions (10), rare joint bleeds (except type 3).

c) Defects of others coagulation factors:

Its frequency is low, except the Factor XI, ranging from 1/500.00 for F VII and 1/2.000.000 for prothrombin (FII) (1). All have autosomal heredity and, unless F XI deficiency, heterozygotes usually have no significant clinical manifestations (1).

d) Hypercoagulable Pictures: Also known as thrombophilia and can be defined as clinical entities with a tendency to hypercoagulability and thrombosis (15), they can be inherited or acquired. Despite the evidence demonstrated in numerous studies in cases of children who suffer strokes, this increased incidence of disorders / thrombogenic factors of hereditary origin, they by themselves cannot fully explain their appearance, because only represent a minor / moderate risk factor (16). All of these entities are listed in  Table 4.

**Table 4:**
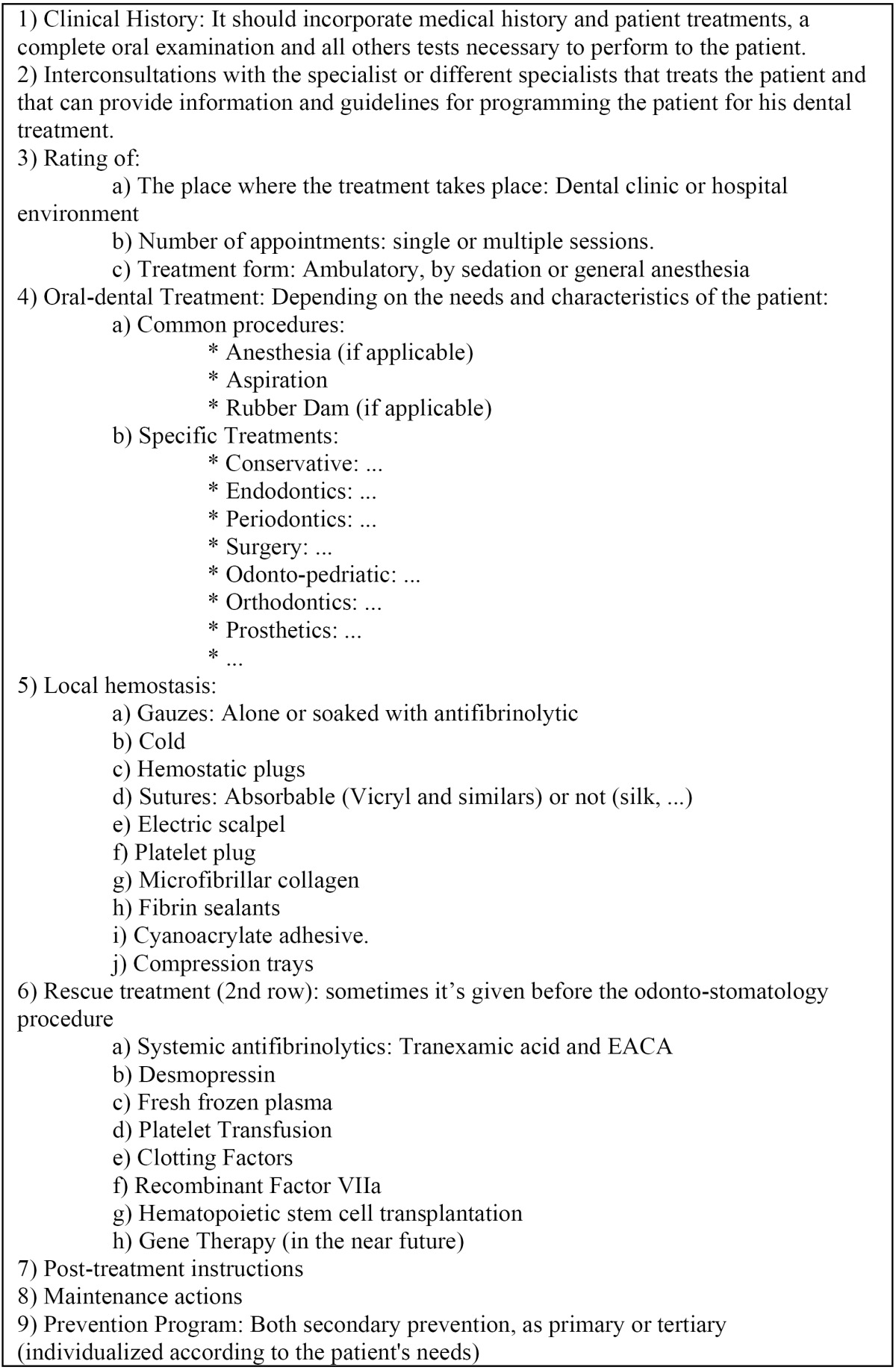
Systematic dental treatment planning. (19-27,30-35).

2) Alterations acquired

A) Current antibody inhibitor of factors: These are disorders that appear after repeated transfusions of factors to patients who lack them. Antibodies are produced by the body against exogenous factors, but may cross-react with proteins of coagulation. They are classified into specific and nonspecific:

B) Deficit of vitamin k: Vitamin K is needed for the processes of synthesis of factors II, VII, IX, and X as well as proteins S and C. Its deficiency causes an increased PT and APTT.

C) Liver diseases: The great importance of the liver in the level of the metabolic processes makes its dysfunction could interfere with the synthesis of vitamin K dependent factors, but there is also decreased factor V and fibrinogen. In addition, there may be thrombocytopenia, platelet dysfunction and primary hyperfibrinolysis. The combination of all this can give rise to different bleeding.

D) Kidney diseases: In nephrotic syndrome, thrombotic processes are described in the renal vein, but also in other vessels, associated with a decrease in antithrombin III, protein S and Factor XIII, on phenomena associated with platelet aggregation. By contrast, in terminal renal failure, there is a tendency to bleeding diathesis.

E) Neoplasias: The medical table may be due to oncotherapy administered to combat the processes, and/or due to the clinical entity in itself. They are mostly, haemorrhagic disorders.

F) Disseminated intravascular coagulation (DIC): In this condition, appears diffuse activation of all phenomena of hemostasis, with consumption of all the factors involved in the process as described above and consequently, the appearance of bleeding disorders that, if kept in time, can cause multi organic fails, and even patient’s death. It can be triggered by multiple factors (germs; neoplasms poisons; tissues damages;) acting self perpetuating the illness, so that treatment, in addition to etiologic should be supportive and substitution of products consumed.

G) Hypercoagulable illnesses: Inside acquired processes that can lead to hypercoagulability tables we could find the presence of antiphospholipid antibodies, anticardiolipin and / or anti-β2-glycoprotein I. It may also occur when increased rates of clotting factors, in hyperhomocysteinemia, prolonged immobilization, aging, surgery, trauma, cancer, obesity, malnutrition, pregnancy, use of oral contraceptives and hormone replacement therapy (15,17).

B) Fibrinolysis pathology:

1) Deficit α2 antiplasmin: Autosomal recessive transmission. Hemorrhages appear because hyperfibrinolytic and homozygous forms are similar to haemophilia. The treatment is done with plasma and antifibrinolytics are used preventively.

2) Deficit inhibitor-1 plasminogen activator: haemorrhagic manifestations appear for lack of control of fibrinolysis and not braking the activity of plasminogen activation factor.

## III Therapeutic attitude of the dentist when facing these patients

When trying to control the bleeding, as well as local hemostasis actions, which we will see later, we can use various products such as:

1) Tranexamic Acid: Acts as antifibrinolytic and is more powerful than the epsilon amino caproic acid (EACA), whereby is rarely used oral level. It can be used as a systemic treatment with a dose of 20 mg / kg body weight or by local rinses, being a good adjuvant in the control of bleeding (18).

2) Desmopressin: It’s a synthetic analogue of the vasopressin hormone, but lacking vasopressor properties. Its administration increases plasma concentrations of F vW and F VIII. The response to the desmopressin is dose-dependent, reaching a maximum effect at 90-120 minutes, and can be administered by intravenous infusion, subcutaneous injection or intranasal spray. Frequent repetition and close in time to administration brings a phenomenon of tachyphylaxis or acute tolerance. Because of this, some authors use it in a single dose, with good results (19).

3) Fresh frozen plasma: Although with prothrombotic supplements we must be very careful, for the risk of vascular obstruction (20), in FV-deficient patients, the infusion of 450 ml of fresh plasma raises the level of the factor 1% to 11% and passed 24 h, it’s maintained a level of 6%. Also the values of TP and the TPPA are modified, passing from 20% to 50% passed 24 h (20).

4) Platelet Transfusions: indicated when the disorder is severe (less than 30,000 platelets) and when other therapeutic measures have not been effective. There is a risk of transmission of infectious diseases and reactions, recommending the platelet transfusion compatible with HLA (21).

5) Recombinant factor VIIa: Its own way of production is not immunogenic in patients with deficiency of factors, or not induce immune response when administered to patients previously sensitized by previous transfusions (patients with inhibitors), having a low thrombogenicity (22).

It’s suitable, in addition to F-deficient patients VII, in haemophiliacs and von Willebrand disease with inhibitors, being in most cases sufficient for a single dose application of the product (23). There are also articles describing their ability to control bleeding in patients with platelet disorders and other bleeding diathesis (24,25). The European Union approved its use in Glanzmann’s thrombasthenia refractory to platelet transfusions in patients with antibodies to GPIIb / IIIa (21).

The systematic assistance for oro-dental treatments for patients with this type of hemostatic disorders is common in many places, although in some tables some protocols or specific products are used.

It’s defined prolonged bleeding after tooth extraction, when it lasts more than three hours after surgery (26). In a patient with disorders of hemostasis, even a routine dental treatment such as a tooth extraction, can cause life-threatening situations (27). So, while in congenital disorders associated with platelet dysfunction, the therapies more often used are platelet transfusions in severe cases and desmopressin and antifibrinolytic drugs in mild cases, it is sometimes necessary to use the factors and alternative therapies, both in the preview preparation of the patient as in the rescue therapies after heavy and / or prolonged bleeding. In these patients, sometimes, it may consider hematopoietic stem cell transplantation as therapeutic tool and perhaps gene therapy will be the future for them (11,28). Also in certain acquired illnesses (as in the acquired von Willebrand disease) treatment may be as in the hereditary ones (29). In these type of patients, when planning a oral-dental treatment plan, it’s necessary to assess the risk of bleeding and the pathology suffered by the patient. If it’s necessary a replacement or prophylactic therapy, the hematologist will be the one, once informed by the dentist, who has to design the preparation protocol of patient’s hemostasis.

Based on what it’s said above, as a systematic care for these patients, it’s proposed:

1. Clinical history: Medical and dental very exhaustive, with exploration and further tests for doing the treatment plan.

2. Internal consultation to the specialist (hematologist / internist): It must be told, in plain language, all the planned treatment and the bleeding complications that may arise (by the type of treatment, oral health status and prior health degree, if the location of the procedure will be unique or it’s going to be done in several areas and the degree of contiguity between them). Based on the information that we provide him, the hematologist will perform the treatment planning pre, intra and post operative required by the patient.

3. Considering the style of treatment (ambulatory, outpatient sedation or under general anesthesia), where doing it and if there is possibility to do it all in one session: In case of require general anesthesia or factors infusion, platelets or any type of blood derivatives, will be held in a hospital environment. Sometimes, it can be performed on an ambulatory way, but always in intimate coordination with the hematology service. Doing everything in one session has the advantage of saving some cost of the complete treatment versus doing it in different sessions, but against this, there’s a several potential risk of haemorrhagic from different focus, and make a mutual decision in agreement with the hematologist. In case of performing under general anesthesia, intubation track is also subject to dispute, some authors prefers nasotracheal technique (18) and others oral one (27), although both have the risk of submucosal hemorrhages in areas not accessible to visual inspection of the dentist, with danger of tracheobronchial aspiration.

4. Performing dental treatment: Except in rare circumstances, the first therapeutic risk actions will be the implementation of anesthesia to the patient. If it’s suggested, prevent nerve block anesthesia techniques, (in particular the inferior alveolar) if feasible, by the possible appearance of bruising in the areas of infiltration. Intraligamentous, intrapulpal, interpapillary or inflitratives anesthesia techniques are recommended in areas close to the periosteum and using anesthesia with vasoconstrictor, which will help to control bleeding. Once treatment begins, we must take precautions to make it the less traumatic treatments as possible, therefore:

a) In odontopediatric treatments:

- Not overused the instrumental in pulpotomy and pulpectomies

- Do not invade gums (supragingival pediatric dental crown)

- Cleaning carefully if there’s granulation tissue under the root for a better control of bleeding

b) In relation to aspiration:

- Support the nozzle on rollers or gauze and never directly on mucous

- Using spit ejector suction instead of surgical suction for being less traumatic.

c) According to the use of the rubber dam, there’s some authors that recommend it (27) because:

- Produces gingival retraction and field isolation

- Increases the operative visibility 

- Reduces the possibility of injuring lips and mucous membranes.

Since staples can be very traumatic, it’s recommendable to maintain the rubber dam with rubber cord pieces introduced between interdental space, if possible, and do not use wood shims for this technique.

d) In orthodontic treatments, bands, brackets and fixed maintainers, always cemented in a supragingival way.

e) Remember that early restorations prevent subsequent extractions, preferring pulpal protections than endodontics ones, and being careful with these last ones to do not overuse the instrumental in the apex. Extractions are the last option.

f) In the case of doing extractions, though there are articles that don’t find advantages to suture wounds, facing not doing it (30), there’s some authors (27) that recommend doing them with atraumatic needles, absorbable sutures and giving the least possible number of stitches.

5. Application of topical hemostasis ways and local measures: There are many possibilities and it’s often the combination of more than one way:

a) Autologous platelet plug associated with systemic tranexamic acid (31)

b) Desmopressin associated with topical tranexamic acid (mouthwashes) (32)

c) Surgicell® and wound closure (25)

d) Pressure with gauze soaked in tranexamic acid besides hemostatic plugs, associated with fibrin sealants, compression splints and antifibrinolytic rinses (33)

e) Surgicell or cellulose plugs or fibrin sponge or microfibrillar collagen or fibrin or cyanoacrylate adhesive, what means, material to fill the socket and antifibrinolytic rinses (27)

f) Splints: Using vacuum splints made this controversial because some authors recommend them (34) while others criticize them (35) because they retain debris and detritus, which can lead to septic processes in the area.

6. Rescue treatment or 2nd row treatment: When all the actions seen above fail, we must appeal to other type of treatment. Normally, it’s usually done with blood derivates or recombinant factor, but always bearing in mind the circumstances of each patient (presence of inhibitors, etc.).

7. Scheduled instruction until post-treatment revision: Until the patient revision, including:

a) Diet: Cold liquid is recommended for 1-2 days after surgery and then soft diet until the removal of sutures and / or revision

b) Analgesia: Evite NSAIDs and administer paracetamol 15 mg / kg every 6 h (18,27) Codeine can also be given, alone or in combination.

c) Desmopressin: If needed, a dose of 2 mg / kg at 12 and 24 h. post treatment (18)

d) Antibiotics: To prevent over infection of the area, which could cause late bleeding (27)

8. Preventive and maintenance programs of oral hygiene: The hygiene of patients with haemophilia and von Willebrand disease is worse than the hygiene of other patients, attributed to fear of bleeding from tooth brushing (13). This can be extrapolated to patients with other types of disorders of hemostasis. However, the periodontal status of patients with von Willebrand disease who come regularly to dental examination is within the considered acceptable parameters (13). Preventive programs in this type of patient, should consider (27,33):

a) Vaccination against hepatitis B

b) Don’t take NSAIDs

c) Maintaining proper oral hygiene

d) Correction of iron deficiency

e) If the patient can rinse, use chemical control of plaque

f) Diagnosis and prenatal counseling in families with a background history.

9) Other circumstances: In patients with haemophilia, it has been observed that the emotional and psychological problems contribute to the onset of spontaneous bleeding (36). Similarly, some authors (1) reported that prolonged oral bleeding and for an extended period of time causes that hemosiderin and other degradation products of hemoglobin deposits on tooth structures, giving the tooth a dark discoloration.

## References

[1] Adeyemo TA, Adeyemo WL, Adediran A, Akinbami AJ, Akanmu AS (2011). Orofacial manifestations of hematological disorders: anemia and hemostatic disorders. Indian J Dent Res.

[2] Murphy WG, Davies MJ, Eduardo A (1993). The haemostatic response to surgery and trauma. Br J Anaesth.

[3] Kaushansky K (2008). Historical review: megakaryopoiesis and thrombopoiesis. Blood.

[4] Jurk K, Kehrel BE (2005). Platelets: physiology and biochemistry. Semin Tromb Hemost.

[5] Gibbins JM (2004). Platelet adhesion signaling and the regulation of thrombus formation. J Cell Sci.

[6] Savage B, Cattaneo M, Ruggeri ZM (2001). Mechanisms of platelets aggregation. Curr Opin Hematol.

[7] Pérez-Gómez F, Bover R (2007). [The new coagulation cascade and its possible
influence on the delicate balance between thrombosis and hemorrhage]. Rev Esp Cardiol.

[8] Seegmiller A, Sarode R (2007). Laboratory Evaluation of platelet function. Hematolog Oncol Clin N Am.

[9] Dyszkiewicz-Korpanty AM, Frenkel EP, Sarode R (2005). Approach to assessment of platelet function: comparison between optical-based platelet-rich plasma and impedence-based whole blood platelet aggregation methods. Clin Appl Thromb Hemost.

[10] Eleftheriou D, Dillon MJ, Brogan PA (2009). Advances in childhood vasculitis. Curr Opin Rheumatol.

[11] Valera MC, Kemoun P, Cousty S, Die P, Payrastre B (2013). Inherited platelet disorders and oral health. J Oral Pathol Med.

[12] Nurden AT, Freson K, Seligsohn U (2012). Inherited platelet disorders. Haemophilia.

[13] Benito-Urdaneta M, Benito-Urdaneta M, Ferrara-Mendez V, Bernardoni-Socorro C, Arteaga-Vizcaíno M (2008). Evaluating periodontal conditions in patients with von Willebrand's disease in Hospital Universitario de Maracaibo (University Hospital, Maracaibo)-Venezuela. Med Oral Patol Oral Cir Bucal.

[14] Kadir RA, Sharief LA, Lee CA (2012). Inherited bleeding disorder in older women. Maturitas.

[15] Favaloro EJ, McDonald D, Lippi G (2009). Laboratory investigation of thrombophilia: the good, the bad and the ugly. Semin Thromb Hemost.

[16] Zadro R, Herak DC (2012). Inherited prothrombotic risk factors in children with first ischemic stroke. Biochem Med (Zagreb).

[17] Anderson JA, Weitz JI (2011). Hypercoagulable states. Crit Care Clin.

[18] Bornert F, Clauss F, Gros CI, Faradji A, Schmittbuhl M, Manière MC (2011). Hemostatic management in pediatric patients with type I von Willebrand disease undergoing oral surgery: case report and literature review. J Oral Maxillofac Surg.

[19] Sánchez-Luceros A, Meschengieser SS, Woods AI, Chuit R, Turdó K, Blanco A (2010). Biological and clinical response to desmopressin (DDAVP) in a retrospective cohort study of children with low von Willebrand factor levels and bleeding history. Thromb Haemost.

[20] Kitamura A, Yamashita H, Okumura T, Asahina I (2011). Extraction of four wisdom teeth in a patient with congenital factor V deficiency haemophilia. Oral Surg Oral Med Oral Pathol Oral Radiol Endod.

[21] Geddis AF (2013). Inherited thrombocytopenias an approach to diagnosis and management. Int Jnl Lab Hem.

[22] Croom KF, McCormack PL (2008). Recombinant factor VIIa (eptacog alfa): a review of its use in congenital hemophilia with inhibitors, acquired hemophilia, and other congenital bleeding disorders. BioDrugs.

[23] Boadas A, Fernandez-Palazzi F, De Bosch NB, Cede-o M, Ruiz-Sáez A (2011). Elective surgery in patients with congénital coagulopathies and inhibitors: experience of the national Haemophilia Centre of Venezuela. Haemophilia.

[24] Weeterings C, Lisman T, de Groot PG (2008). Tissue factor-independence effects of recombinant factor VIIa on hemostasis. Semin Hematol.

[25] Hers I, Mumford A (2008). Understanding the therapeutic action of recombinant factor VIIa in platelet disorders. Platelets.

[26] Plugg I, Mauser-Bunschoten EP, Bröker-Vriends AHJT, Ploos van Amstel HK, van der Booms JG, van Diemen-Homan JEM (2006). Bleeding in carriers of haemophilia. Blood.

[27] Rayen R, Hariharan VS, Elavazhagan N, Kamalendran N, Varadarajan R (2011). Dental management of haemophiliac child under general anesthesia. J Indian Soc Pedod Prev Dent.

[28] Nurden P, Nurden AT (2008). Congenital disorders associated with platelet dysfunctions. Thromb Haemost.

[29] Lison S, Dietrich W, Spannagl M (2012). Review article: unexpected bleeding in the operating room: the role of acquired von Willebrand disease. Anesth Analg.

[30] Jover-Cerveró A, Poveda-Roda R, Bagan JV, Jiménez-Soriano Y (2007). Dental treatment of patients with coagulation factors alterations: an update. Med Oral Patol Oral Cir Bucal.

[31] Nurden P, Youlouz-Marfak I, Siberchicot F, Kostrzewa E, Andia I, Anitua E (2011). Use of autologous platelet-rich clots for prevention of local injury bleeding in patients with severe inherited mucocutaneous bleeding disorders. Haemophilia.

[32] Nickles K, Wohlfeil M, Alesci S, Miesbach W, Eickholz P (2010). Comprehensive treatment of periodontitis in patients with von Willebrand disease. J Periodontol.

[33] Seligsohn U (2012). Treatment of inherited platelet disorders. Haemophilia.

[34] Israels S, Schwetz N, Boyar R, McNicol A (2006). Bleedings disorders: Characterization, dental considerations and managements. J Can Dent Assoc.

[35] Kumar NJ, Kumar RA, Varadarajan R, Sharma N (2007). Specialty dentistry for the haemophiliac: is there a protocol in place?. Indian J Dent Res.

[36] Chiono O (1968). Dental anesthesia for the hemophilic patient. Anesth Prog.

